# Dynamics of HDL-Cholesterol Following a Post-Myocardial Infarction Cardiac Rehabilitation Program

**DOI:** 10.31083/RCM25399

**Published:** 2025-01-13

**Authors:** Carlos Bertolín-Boronat, Héctor Merenciano-González, Víctor Marcos-Garcés, María Luz Martínez-Mas, Josefa Inés Climent Alberola, Nerea Pérez, Laura López-Bueno, María Concepción Esteban-Argente, María Valls Reig, Ana Arizón Benito, Alfonso Payá Rubio, César Ríos-Navarro, Elena de Dios, Jose Gavara, Juan Sanchis, Vicente Bodi

**Affiliations:** ^1^Department of Cardiology, Hospital Clinico Universitario de Valencia, 46010 Valencia, Spain; ^2^INCLIVA Health Research Institute, 46010 Valencia, Spain; ^3^Network Biomedical Research Center for Cardiovascular Diseases (CIBER-CV), 28029 Madrid, Spain; ^4^Department of Rehabilitation, Hospital Clinico Universitario de Valencia, 46010 Valencia, Spain; ^5^Hospital Clinico Universitario de Valencia, 46010 Valencia, Spain; ^6^Centre for Biomaterials and Tissue Engineering, Universitat Politècnica de València, 46022 Valencia, Spain; ^7^Department of Medicine, Faculty of Medicine and Odontology, University of Valencia, 46010 Valencia, Spain

**Keywords:** HDL-cholesterol, cardiac rehabilitation, myocardial infarction, secondary prevention

## Abstract

**Background:**

Exercise-based cardiac rehabilitation programs (CRP) are recommended for patients following acute coronary syndrome to potentially improve high-density lipoprotein cholesterol (HDL-C) levels and prognosis. However, not all patients reach target HDL-C levels. Here we analyze the dynamics and predictors of HDL-C increase during CRP in patients following ST-segment elevation myocardial infarction or occlusion myocardial infarction.

**Methods:**

We conducted a prospective study of myocardial infarction patients who completed exercise-based Phase 2 CRP. Data was collected on clinical variables, cardiovascular risk factors, treatment goals, pharmacological therapy, and health outcomes through questionnaires at the beginning and at the end of Phase 2 CRP. Lipid profile analysis was performed before discharge, 4 to 6 weeks after discharge, and at the end of Phase 2 CRP. Changes in lipid profiles were evaluated, and predictors of failure to increase HDL-C levels were identified by binary logistic regression analysis.

**Results:**

Our cohort comprised 121 patients (mean age 61.67 ± 10.97 years, 86.8% male, and 47.9% smokers before admission). A significant decrease in total cholesterol, triglycerides, and low-density lipoprotein cholesterol (LDL-C) were noted, along with an increase in HDL-C (43.87 ± 9.18 vs. 39.8 ± 10.03 mg/dL, *p* < 0.001). Patients achieving normal HDL-C levels (>40 mg/dL in men and >50 mg/dL in women) significantly increased from 34.7% at admission to 52.9% the end of Phase 2. Multivariable analysis revealed smoking history (hazard ratio [HR] = 0.35, 95% confidence interval [CI], 0.11–0.96, *p* = 0.04), increased reduction in total cholesterol (HR = 0.94, 95% CI, 0.89–0.98, *p* = 0.004), and increased reduction in LDL-C (HR = 0.94, 95% CI, 0.89–0.99, *p* = 0.01) were inversely associated with failure to increase HDL-C levels. Conversely, higher HDL-C before CRP (HR = 1.15, 95% CI, 1.07–1.23, *p* < 0.001) and increased lipoprotein (a) (HR = 1.01, 95% CI, 1–1.02, *p* = 0.04) predicted failure to increase HDL-C levels. No significant correlations were found with Mediterranean diet adherence, weekly physical activity, training modalities, or physical fitness parameters.

**Conclusions:**

Participation in an exercise-based Phase 2 CRP led to mild but significant increases in HDL-C. Smoking history and patients experiencing substantial reductions in total cholesterol and LDL-C were more likely to experience HDL-C increases, unlike those with higher HDL-C and lipoprotein (a) levels before CRP.

## 1. Introduction

Coronary artery disease (CAD) is the leading cause of morbidity and mortality in the developed world [1, 2], with acute coronary syndrome (ACS) often serving as the initial manifestation of the disease. Once diagnosed, ACS patients are classified as very high-risk individuals, necessitating stringent efforts to reach secondary prevention targets for cardiovascular risk factors [3, 4]. Cardiac rehabilitation programs (CRP) are strongly recommended for these patients, supported by the highest level of evidence [2, 3]. Not only do CRPs improve perceived quality of life and long-term prognosis, but they also facilitate more effective control of cardiovascular risk factors including lipid profiles, smoking habits, and the adoption of a healthy lifestyle [5, 6, 7].

Lipid control, particularly for low-density lipoprotein cholesterol (LDL-C), is a critical target for secondary prevention due to its causal role in the atherosclerotic process [8, 9]. Beyond LDL-C-centric recommendations, the guidelines also provide recommendations for secondary target goals. For instance, high levels of triglycerides (defined as those above 150 mg/dL) can increase cardiovascular risk [6]. Low levels of high-density lipoprotein cholesterol (HDL-C) have also been associated with increased risk of atherosclerotic cardiovascular events [10, 11, 12].

In recent years, the role of HDL-C in cardiovascular risk has undergone significant scrutiny. Studies now suggest that the functional aspects and individual components of HDL-C may offer greater clinical insights than its concentration levels in blood serum [13, 14, 15, 16]. Nevertheless, the role of HDL-C as a biomarker for cardiovascular risk is extensively documented in the scientific literature [17]. A recent study has revealed a U-shaped relationship between HDL-C and cardiovascular risk, suggesting that both very low and very high levels of HDL-C are associated with increased risk [18]. This observation underscores the potential benefits of interventions aimed at maintaining HDL-C within an optimal range, particularly since many patients do not reach the necessary HDL-C targets following an ACS diagnosis [7]. However, the evidence supporting therapeutic interventions specifically aimed at increasing HDL-C remains limited.

The aim of our study is to assess the impact of an exercise-based Phase 2 CRP on HDL-C dynamics in patients following ST-segment elevation myocardial infarction (STEMI) or occlusion myocardial infarction (OMI). We aim to analyze the proportion of patients who attain the recommended HDL-C levels following Phase 2 CRP and to identify predictors of HDL-C increase during the intervention. This analysis will provide insights into the effectiveness of exercise as a component of cardiac rehabilitation in modifying lipid profiles, particularly focusing on HDL-C as a critical factor in cardiovascular risk management.

## 2. Materials and Methods

### 2.1 Population

**Fig. 1.**
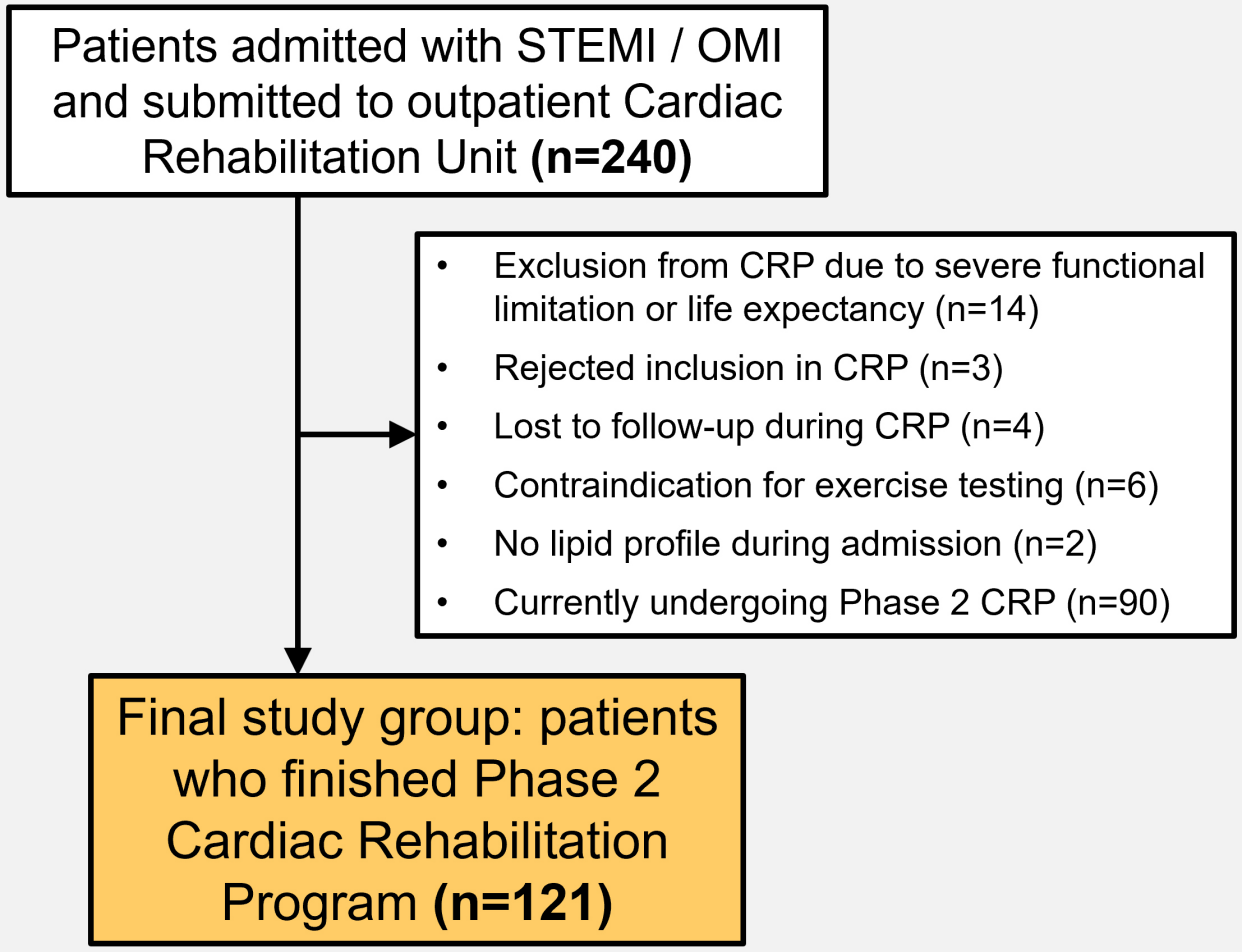
**Patient selection process and exclusion criteria**. Abbreviations: CRP, cardiac rehabilitation program; OMI, occlusion myocardial infarction; STEMI, ST-segment elevation acute myocardial infarction.

Our study is derived from a prospective cohort of STEMI or OMI patients referred to the CRP in our institution, which is a high-complexity tertiary care hospital. All STEMI/OMI patients were referred to the CRP, unless contraindicated or the patient rejected treatment. We considered patients who completed Phase 2 CRP between January 2022 and April 2024 for inclusion. Exclusion criteria were severe functional limitation or life expectancy (n = 14), voluntary rejection of CRP (n = 3), follow-up losses during CRP (n = 4), contraindication for exercise testing (n = 6), or unavailable lipid profile during admission (n = 2). The patient selection flowchart is depicted in Fig. 1.

Baseline clinical characteristics registered include sex, cardiovascular risk factors, infarct location, Killip class during admission, Global Registry of Acute Coronary Events (GRACE) risk score, and echocardiographic left ventricular ejection fraction (LVEF) before discharge.

### 2.2 Cardiac Rehabilitation Program

Our local CRP offers a Phase 2 program to STEMI and OMI patients after hospital discharge. Follow-up is provided by a multidisciplinary team of cardiologists, physical medicine and rehabilitation physicians, trained nurses, and physiotherapists. Individualized ambulatory aerobic and strength training is provided after conventional or cardiopulmonary exercise testing (C/CPET), and in higher-risk patients during 8 to 20 sessions of in-hospital supervised training. During ambulatory training, patients were instructed to perform 3 to 5 weekly sessions of aerobic training (30 to 60 minutes per session) with increasing workload, according to individualized Karvonen targets (40 to 80% of heart rate reserve; moderate to high intensity), as well as 2 to 3 weekly individualized strength training sessions. In-hospital supervised 1-hour sessions consisted of warming-up (5 to 10 minutes), aerobic training in cycle ergometers with incremental workload (20 to 35 minutes of continuous or high-intensity interval training), strength training (10 to 15 minutes), and cool-down exercises. A multicomponent lifestyle intervention plan including patient education and empowerment was performed by the team, along with pharmacological therapy modifications to achieve optimal control of cardiovascular risk factors and other treatment targets (e.g., prognostic and symptomatic therapy for heart failure patients). At the end of Phase 2, after a median 5.55 (4.59–7.03) months, C/CPET was repeated, with updated training guidance for Phase 3 provided, and analysis of achievement of control goals.

### 2.3 Health Outcomes after Phase 2 CRP

As part of the integral evaluation during Phase 2 CRP, several factors were analyzed at the beginning and at the end of the intervention. These include smoking cessation, reduction of systolic blood pressure (in mmHg), optimal LDL-C goal (defined as less than 55 mg/dL) and target glycated hemoglobin (HbA1c) in diabetic patients (defined as less than 7%) were registered. Weight reduction (absolute -in kg- and relative) and changes in body mass index (BMI) were analyzed, as well as noting BMI below 30 (defining non-obese patients). Specific questionnaires were used to assess adherence to Mediterranean diet (PREvención con DIeta MEDiterránea, PREDIMED; good adherence was defined as greater than or equal to 8 points), therapeutic adherence to pharmacological therapy (Morisky-Green; good adherence was defined as 4 points), quality of life (36-Item Short Form Survey Instrument, SF-36), depression (Patient Health Questionnaire 2-item, PHQ2), and anxiety (Generalized Anxiety Disorder 2-item, GAD2). We also assessed weekly physical activity as self-reported by patients in a specific questionnaire (International Physical Activity Questionnaire Short Form, IPAQ). Metabolic equivalents (METS) per week were estimated accounting for 3.3 METS per minute of low-intensity activity, 4 METS per minute of moderate-intensity activity, and 8 METS per minute of high-intensity activity [19].

Finally, C/CPET were used to analyze the change in peak oxygen consumption (VO2) (absolute -in mL/kg/min- and relative), measured by cardiopulmonary or estimated by conventional exercise testing. METS reached during conventional exercise testing were automatically calculated by the ergometer using the following formula:

$$\begin{array}{c} \text{ M E T S}=\frac{\left(\text{ speed }\times \text{ 0.1 }\right)+\left(\text{ treadmill grade }/\text{ 100}\times \text{ 1.8}\times \text{ speed }\right)+\text{3.5}}{\text{3.5}} \end{array}$$

In this instance, speed was expressed as m per min and treadmill grade as percent. The METS value is interpolated between two stages. Predicted functional capacity was calculated using the reference equation from the Fitness Registry and the Importance of Exercise National Database (FRIEND) registry [20].

### 2.4 Lipid-Lowering Therapy

We registered the lipid-lowering therapy that patients received at the first visit at our CRP, i.e., the lipid-lowering therapy which was prescribed at discharge by referring physicians, and the lipid-lowering therapy that patients were receiving at the end of Phase 2. Drugs and doses were registered and categorized as: statins, ezetimibe, bempedoic acid, fibrates, and pro-protein convertase subtilisin/kexin type 9 (PCSK9) inhibitors.

### 2.5 Blood Testing and Lipid Profile Analysis

Our local protocol mandates that all patients must undergo an initial blood test, which includes a complete lipid and metabolic profile, during their admission. A subsequent blood test is conducted early after hospital discharge, typically between 4 to 6 weeks. Additionally, during Phase 2 CRP further blood tests are conducted as deemed clinically necessary by the attending cardiologists. The most recent blood test conducted before the end of Phase 2 was considered the final assessment, and its results were compared with values obtained at admission and shortly after discharge.

The parameters recorded included fasting glucose (mg/dL), HbA1c (in %), total cholesterol (mg/dL), triglycerides (mg/dL), HDL-C (mg/dL), LDL-C (mg/dL), and non-HDL-C (mg/dL). Additionally, lipoprotein (a) levels were analyzed during admission or at the first ambulatory blood test.

### 2.6 HDL-C Dynamics and Targets

We analyzed the proportion of patients with low (defined as ≤40 mg/dL in men and ≤50 mg/dL in women) and normal (defined as >40 mg/dL in men and >50 mg/dL in women) HDL-C levels at admission, early after discharge, and at the end of Phase 2 CRP [12]. Patients with at least a 1 mg/dL increase in HDL-C levels between admission and the end of Phase 2 CRP were considered to have increased their HDL-C levels during the CRP.

### 2.7 Statistical Analysis

The one-sample Kolmogorov–Smirnov test was used to test normal data distribution. For continuous parametric variables data were expressed as mean ± standard deviation and were analyzed with Student’s *t* test. Continuous non-parametric variables are shown as median plus interquartile range and compared using the Mann–Whitney U test. Qualitative variables are presented as percentages and compared using the chi-square test or Fisher’s exact test. Student’s *t* test for paired samples was used to compare HDL-C and other lipid profile levels between admission, early after discharge, and at the end of Phase 2 CRP.

Univariate analyses were performed to identify variables associated with HDL-C increase after Phase 2 CRP. Variables with *p*-value < 0.1 in univariate analysis were included as cofactors in a multivariate binary logistic regression model. The predicted probability in the final binary logistic regression model was used to predict HDL-C increase after Phase 2 CRP. Receiver operating characteristic (ROC) curves were computed to independently analyze the discrimination ability the model. An area under the ROC curve (AUC) >0.8 was considered excellent [13]. Statistical significance was considered for 2-tailed *p*-values < 0.05. The SPSS statistical package version 26.0 (IBM Corp., Armonk, NY, USA) was used.

## 3. Results

### 3.1 Cohort Description

The final study group comprised 121 patients who completed Phase 2 CRP following a STEMI or OMI, who received treatment at our institution. Baseline characteristics of the cohort are depicted in Table 1. The majority were middle-aged (mean age 61.67 ± 10.97 years), male (86.8%), and presenting with anterior (47.9%) or inferior (43.8%) STEMI. Ambulatory training was the primary intervention for most patients (n = 100, 82.6%), while 21 patients (17.4%) participated in additional supervised in-hospital training sessions. The mean LVEF was 53.12 ± 10.79%, and 39 (32.2%) patients had LVEF below 50% at discharge.

**Table 1.** **Baseline characteristics of patients stratified by HDL-C levels following Phase 2 CRP cardiac rehabilitation post-myocardial infarction**.

			All patients (n = 121)	Increase in HDL-C (n = 90)	No increase in HDL-C (n = 31)	*p*-value
Clinical variables						
	Age (years)		61.67 ± 10.97	61.73 ± 10.76	61.49 ± 11.72	0.92
	Male sex (%)		105 (86.8)	77 (85.6)	28 (90.3)	0.499
	Hypercholesterolemia (%)		106 (87.6)	79 (87.8)	27 (87.1)	0.921
	Hypertension (%)		66 (54.5)	49 (54.4)	17 (54.8)	0.97
	Diabetes mellitus (%)		26 (21.5)	20 (22.2)	6 (19.4)	0.737
	Killip class ≥2 (%)		34 (28.1)	27 (30)	7 (22.6)	0.428
	GRACE risk score		109 [97–132.5]	111.5 [96.75–134.63]	108 [97–130]	0.831
	Infarct location					0.635
		Anterior (%)	58 (47.9)	43 (47.8)	15 (48.4)	
		Inferior (%)	53 (43.8)	38 (42.2)	15 (48.4)	
		Lateral (%)	7 (5.8)	6 (6.7)	1 (3.2)	
		OMI (%)	3 (2.5)	3 (3.3)	0 (0)	
	LVEF (%)		53.12 ± 10.79	51.81 ± 11.17	56.9 ± 8.7	0.011
	LVEF <50% (%)		39 (32.2)	35 (38.9)	4 (12.9)	0.008*
	Exercise training modality during CRP					0.733
		Ambulatory training (%)	100 (82.6)	75 (83.3)	25 (80.6)	
		Supervised in-hospital training (%)	21 (17.4)	15 (16.7)	6 (19.4)	
Cardiovascular risk factors						
	Smoking habit before CRP (%)		58 (47.9)	50 (55.6)	8 (25.8)	0.004
	Smoking habit after CRP (%)		7 (5.8)	6 (6.7)	1 (3.2)	0.479
	Systolic pressure before CRP (mmHg)		125.94 ± 16.13	126.56 ± 16.73	124.16 ± 14.34	0.478
	Systolic pressure after CRP (mmHg)		115.52 ± 10.35	115.31 ± 10.31	116.13 ± 10.62	0.706
	Mean change in systolic pressure (mmHg)		–10.42 ± 13.52	–11.24 ± 13.49	–8.03 ± 13.54	0.256
	Total cholesterol before CRP (mg/dL)		163.26 ± 43.97	155.2 ± 38.12	186.65 ± 51.6	0.003
	Total cholesterol after CRP (mg/dL)		103.1 ± 19.18	102.27 ± 19.33	105.52 ± 18.87	0.415
	Mean change in total cholesterol (mg/dL)		–60.16 ± 42.76	–52.93 ± 38.34	–81.13 ± 48.41	0.001
	Triglycerides before CRP (mg/dL)		127 [99.5–170]	121 [98.5–178.5]	133 [102–164]	0.863
	Triglycerides after CRP (mg/dL)		91 [65–126]	91 [65–127.5]	87 [68–121]	0.715
	Mean change in triglycerides (mg/dL)		–40.38 ± 61.51	–39.82 ± 62.49	–42 ± 59.53	0.866
	HDL-C before CRP (mg/dL)		39.8 ± 10.03	36.84 ± 8.14	48.39 ± 10.18	<0.001
	HDL-C after CRP (mg/dL)		43.87 ± 9.18	44.51 ± 9.23	42 ± 8.9	0.185
	Mean change in HDL-C (mg/dL)		4.07 ± 8.78	7.67 ± 6.78	–6.39 ± 4.44	<0.001^#^
	LDL-C before CRP (mg/dL)		102.58 ± 35.49	97.01 ± 30.99	118.74 ± 42.73	0.003
	LDL-C after CRP (mg/dL)		44.71 ± 14.13	43.33 ± 14.68	48.71 ± 11.71	0.113
	Mean change in LDL-C (mg/dL)		–57.87 ± 35.95	–53.68 ± 32.12	–70.03 ± 43.63	0.028
	Lipoprotein (a) (mg/dL)		50.71 ± 47.32	44.85 ± 41.13	67.9 ± 59.56	0.056
	LDL-C <55 mg/dL after CRP (%)		100 (82.6)	76 (84.4)	24 (77.4)	0.373
	Weight before CRP (kg)		80.65 ± 15.89	80.19 ± 15.26	81.98 ± 17.79	0.591
	Weight after CRP (kg)		78.46 ± 14.86	78.48 ± 15.09	78.38 ± 14.44	0.975
	Absolute (kg) and % change in weight		–2.19 ± 6.2, –2.32 ± 7.36%	–1.71 ± 5.55, –1.9 ± 6.63%	–3.6 ± 7.72, –3.51 ± 9.18%	0.145, 0.296
	BMI before CRP		27.68 ± 4.89	27.33 ± 4.53	28.7 ± 5.77	0.178
	BMI after CRP		26.9 ± 4.5	26.67 ± 4.37	27.58 ± 4.88	0.334
	Mean change in BMI		–0.78 ± 2.04	–0.66 ± 1.83	–1.13 ± 2.54	0.273
	BMI ≥30 before CRP (%)		29 (24)	22 (24.4)	7 (22.6)	0.834
	BMI ≥30 after CRP (%)		19 (15.7)	15 (16.7)	4 (12.9)	0.619
	HbA1c <7% after CRP (%)		114 (94.2)	84 (93.3)	30 (96.8)	0.479
	PREDIMED questionnaire (points)		9.96 ± 2.06	9.86 ± 2.11	10.26 ± 1.91	0.35
	PREDIMED questionnaire ≥8 points (%)		107 (88.4)	78 (86.7)	29 (93.5)	0.302
	Therapeutic adherence after CRP (4 points in Morisky-Green)		116 (95.9)	87 (96.7)	29 (93.5)	0.452
Quality of life outcomes						
	SF-36 (mean) before CRP (points)		61.61 ± 20.48	62.14 ± 19.63	60.09 ± 23.05	0.633
	SF-36 (mean) after CRP (points)		71.13 ± 19.98	71.47 ± 18.21	70.17 ± 24.73	0.79
	Mean change in SF-36 (points)		9.52 ± 18.08	9.33 ± 17.5	10.08 ± 19.95	0.843
	PHQ2 before CRP (points)		1.5 ± 1.61	1.33 ± 1.4	1.97 ± 2.07	0.121
	PHQ2 after CRP (points)		1.04 ± 1.39	0.97 ± 1.21	1.26 ± 1.81	0.408
	Mean change in PHQ2 (points)		–0.45 ± 1.52	–0.37 ± 1.42	–0.71 ± 1.77	0.279
	GAD2 before CRP (points)		1.93 ± 1.66	1.82 ± 1.51	2.23 ± 2.05	0.319
	GAD2 after CRP (points)		1.43 ± 1.51	1.48 ± 1.42	1.29 ± 1.76	0.553
	Mean change in GAD2 (points)		–0.5 ± 1.52	–0.34 ± 1.52	–0.94 ± 1.46	0.107
Physical fitness variables						
	IPAQ before CRP (METS/week)		1988.2 ± 2088.67	1960.61 ± 1826.6	2068.31 ± 2673.77	0.806
	IPAQ after CRP (METS/week)		4466.2 ± 2941.46	4293.77 ± 2755.61	4966.82 ± 3425.44	0.274
	Absolute (METS/week) and % change in IPAQ		2478 ± 2968.51, 334.12 ± 572.67%	2333.16 ± 2675.73, 266.43 ± 489.67%	2898.52 ± 3709.81, 521.39 ± 734.29%	0.363, 0.085
	Peak VO2 before CRP (mL/kg/min)		25.02 ± 9.23	24.75 ± 9.41	25.8 ± 8.77	0.588
	Peak VO2 after CRP (mL/kg/min)		29.67 ± 10.97	29.16 ± 11.01	31.15 ± 10.91	0.386
	Absolute (mL/kg/min) and % change in peak VO2		4.65 ± 5.37, 20.46 ± 25.93%	4.41 ± 5.28, 19.85 ± 25.71%	5.35 ± 5.65, 22.24 ± 26.93%	0.401, 0.661
	Predicted functional capacity before CRP (%)		79.76 ± 31.29	78.9 ± 33.05	82.25 ± 25.83	0.609
	Predicted functional capacity after CRP (%)		93.28 ± 31.98	92.01 ± 32.67	96.97 ± 30.09	0.458
	Mean change in predicted functional capacity (%)		13.52 ± 22.97	13.11 ± 24.8	14.72 ± 16.82	0.738
Lipid-lowering therapy before Phase 2 CRP**						
	High-intensity statins (%)		112 (92.6)	84 (93.3)	28 (90.3)	0.582
	Ezetimibe (%)		63 (52.1)	44 (48.9)	19 (61.3)	0.233
	Fibrates (%)		0 (0)	0 (0)	0 (0)	-
	Bempedoic acid (%)		0 (0)	0 (0)	0 (0)	-
	PCSK9 inhibitors (%)		0 (0)	0 (0)	0 (0)	-
Lipid-lowering therapy at the end of Phase 2 CRP**						
	High-intensity statins (%)		112 (92.6)	82 (91.1)	30 (96.8)	0.3
	Ezetimibe (%)		110 (90.9)	80 (88.9)	30 (96.8)	0.188
	Fibrates (%)		1 (0.8)	0 (0)	1 (3.2)	0.087
	Bempedoic acid (%)		11 (9.1)	9 (10)	2 (6.5)	0.553
	PCSK9 inhibitors (%)		16 (13.2)	15 (16.7)	1 (3.2)	0.057

* LVEF was included in multivariate model as a continuous variable. ^#^ As a definitory variable, change in HDL-C was not included in multivariate model. ** Lipid-lowering therapy was not included in multivariate model to avoid indication and protopathic bias. Abbreviations: BMI, body mass index; CRP, cardiac rehabilitation program; GAD2, Generalized Anxiety Disorder 2-item; GRACE, Global Registry of Acute Coronary Events; HbA1c, glycated haemoglobin; HDL-C, high-density lipoprotein cholesterol; IPAQ, International Physical Activity Questionnaire; LDL-C, low-density lipoprotein cholesterol; LVEF, left ventricular ejection fraction; METS, metabolic equivalents; OMI, occlusion myocardial infarction; PCSK9, pro-protein convertase subtilisin/kexin type 9; PHQ2, Patient Health Questionnaire 2-item; PREDIMED, PREvención con DIeta MEDiterránea; SF-36, 36-Item Short Form Survey Instrument; VO2, oxygen consumption.

### 3.2 Health Outcomes after Phase 2 CRP

Significant improvements to cardiovascular risk factors and key parameters were achieved at the conclusion of Phase 2 CRP. Nearly half of the population were smokers prior to admission (47.9%), and smoking cessation was achieved in most cases (87.9% of previous smokers). Furthermore, a mild but significant weight loss was noted (absolute reduction –2.19 ± 6.2 kg, relative reduction –2.32 ± 7.36%, *p* < 0.001). Nearly perfect adherence was observed with the Mediterranean diet (88.4% scored ≥8 points in PREDIMED questionnaire) as well as pharmacological therapy (95.9% scored 4 points in Morisky-Green questionnaire). Additionally, significant improvements were noted in quality of life (+9.52 ± 18.08 points in SF-36 questionnaire, *p <* 0.001). Functional capacity also improved (+4.65 ± 5.37 mL/kg/min/+20.46 ± 25.93%, in peak VO2), and both depression and anxiety symptoms decreased (–0.45 ± 1.52 points, *p* = 0.001 and –0.5 ± 1.52 points, *p* < 0.001 in PHQ2 and GAD2 questionnaires, respectively).

### 3.3 Lipid Profile and HDL-C Dynamics during Phase 2 CRP

**Fig. 2.**
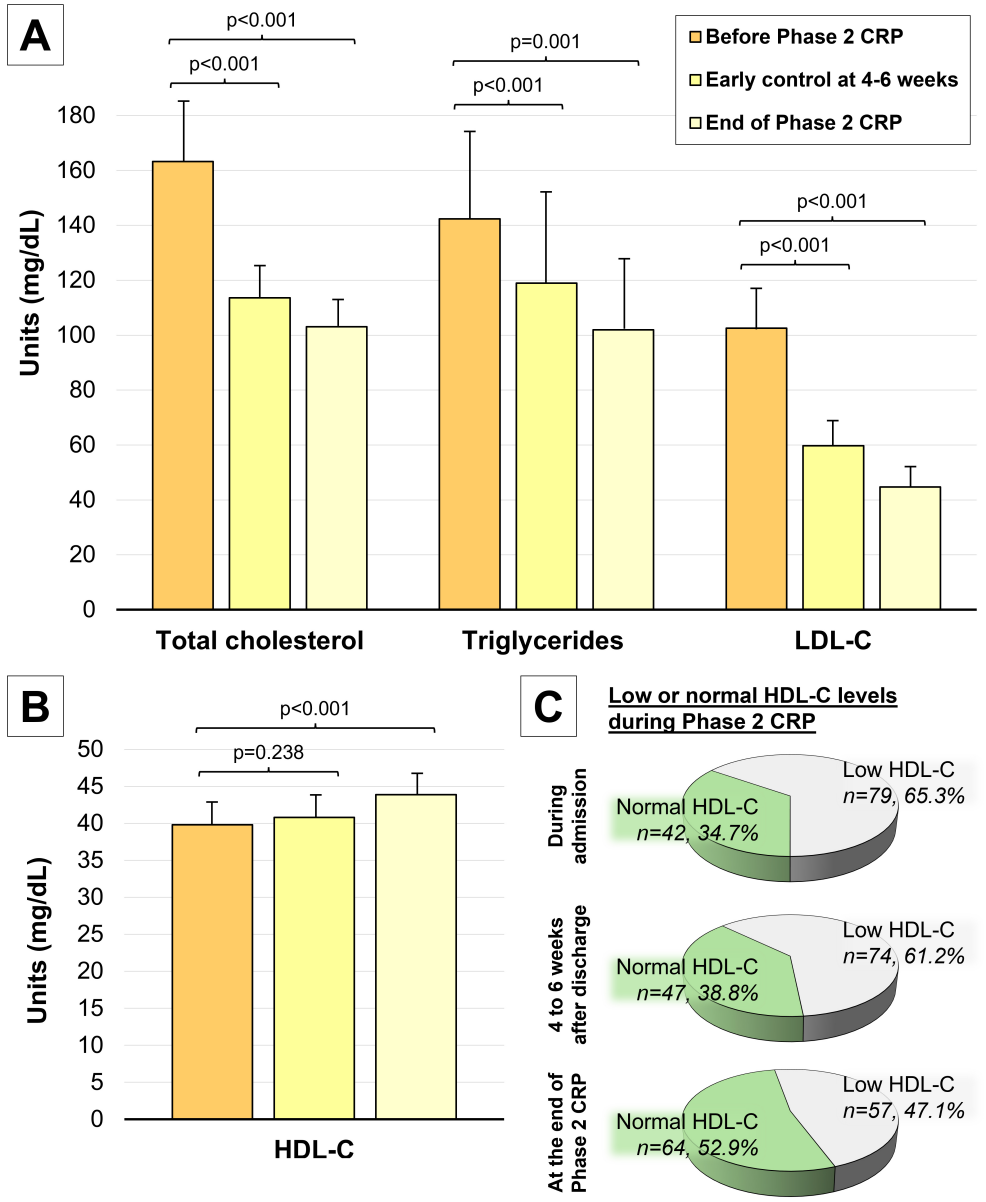
**Changes to the lipid profile during Phase 2 CRP**. (A) Significant decreases in total cholesterol, triglycerides and LDL-C are noted after Phase 2 CRP. (B) Significant increases in HDL-C levels were noted after Phase 2 CRP. (C) The proportion of patients achieving normal HDL-C levels (defined as >40 mg/dL in men and >50 mg/dL in women) significantly increased during Phase 2 CRP. Abbreviations: CRP, cardiac rehabilitation program; HDL-C, high-density lipoprotein cholesterol; LDL-C, low-density lipoprotein cholesterol.

During Phase 2 of the CRP, a significant decrease in total cholesterol, triglycerides, and LDL-C was noted (Fig. 2A). Most patients (n = 100, 82.6%) achieved the target LDL-C level of less than 55 mg/dL by the end of Phase 2. Additionally, HDL-C levels significantly increased during the intervention (43.87 ± 9.18 vs. 39.8 ± 10.03 mg/dL, *p* < 0.001) (Fig. 2B). The proportion of patients reaching normal HDL-C levels also increased from baseline (n = 42, 34.7%) to early control (4 to 6 weeks after discharge, n = 47, 38.8%) and at the conclusion of Phase 2 CRP (n = 64, 52.9%, *p* < 0.001) (Fig. 2C). Furthermore, lipid-lowering therapy was uptitrated in 73.6% of patients (Table 1). The most frequent changes included prescription of ezetimibe, increases in statin equivalent doses, and the addition of PCSK9 inhibitors or bempedoic acid.

### 3.4 Predictors of HDL-C Levels after Phase 2 CRP

**Fig. 3.**
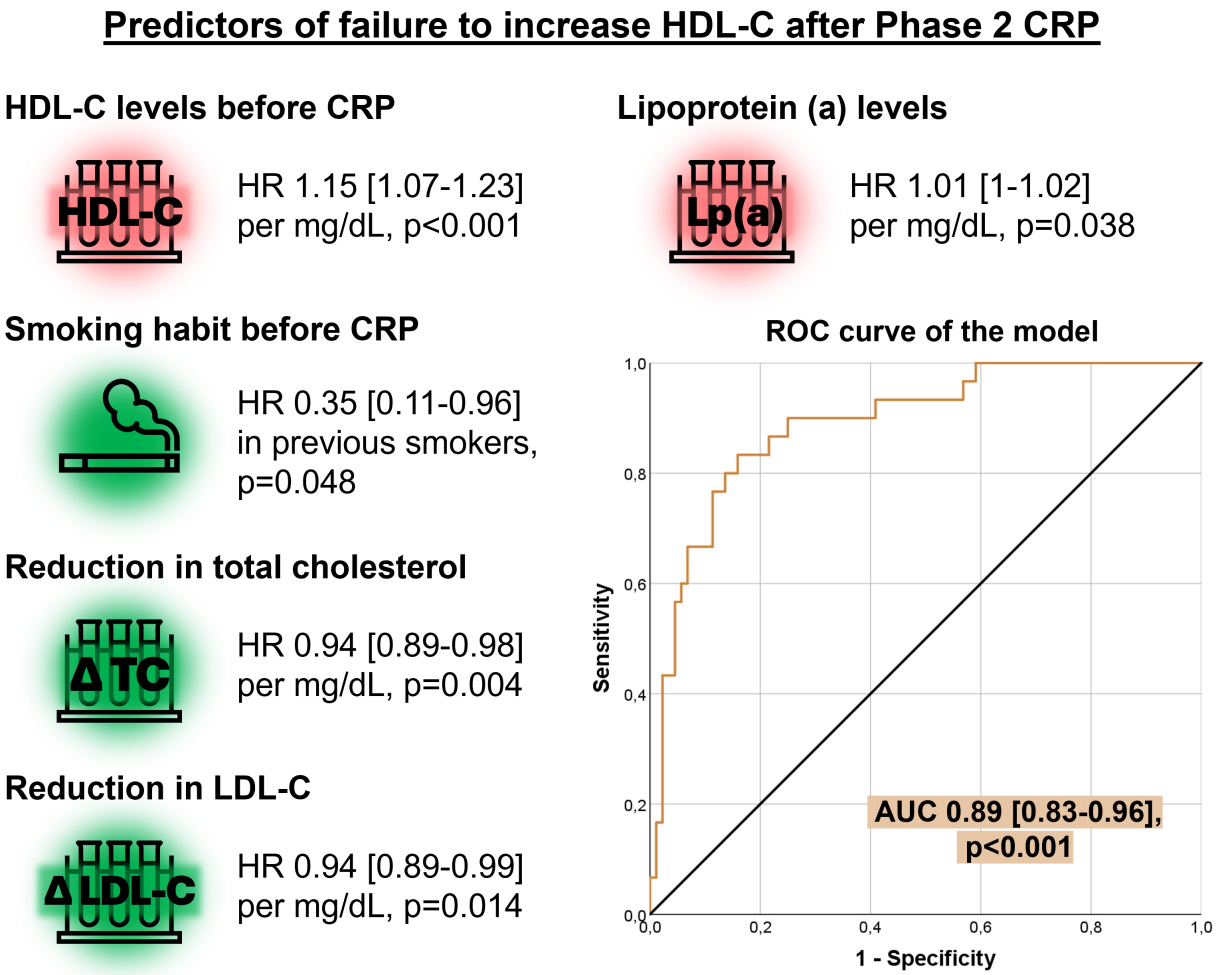
**Multivariate analysis of predictors for failure to increase HDL-C after Phase 2 CRP**. Higher HDL-C and Lp(a) before CRP associated with a lower probability of HDL-C increase after Phase 2. On the contrary, smoking habit before CRP and more pronounced decreases in total cholesterol and LDL-C associated with higher odds of HDL-C increase after Phase 2. Abbreviations: AUC, area under the curve; CRP, cardiac rehabilitation program; HDL-C, high-density lipoprotein cholesterol; HR, hazard ratio; LDL-C, low-density lipoprotein cholesterol; Lp(a), lipoprotein (a); ROC, receiver operating characteristic; TC, total cholesterol.

Multivariable binary logistic regression analysis identified several factors influencing HDL-C levels after Phase 2 CRP (Table 2, Fig. 3). Notably, a history of smoking prior to CRP reduced the likelihood of failing to increase HDL-C levels, or more clearly was associated with an HDL-C increase (hazard ratio [HR] = 0.35, 95% confidence interval [CI], 0.11–0.96, *p* = 0.04). Furthermore, greater reductions in total cholesterol (HR = 0.94, 95% CI, 0.89–0.98, *p* = 0.004) and LDL-C levels (HR = 0.94, 95% CI, 0.89–0.99, *p* = 0.01) were associated with similar improvements to HDL-C levels. In contrast, higher baseline levels of HDL-C before CRP (HR = 1.15, 95% CI, 1.07–1.23, *p* < 0.001) and increased lipoprotein (a) levels (HR 1.01, 95% CI, 1–1.02, *p* = 0.04) were predictive of failure to increase HDL-C levels. The model showed excellent predictive ability, with an AUC of 0.89 (95% CI, 0.83–0.96, *p* < 0.001).

**Table 2.** **Multivariate analysis of predictors for failure to increase HDL-C levels after Phase 2 CRP post-myocardial infarction**.

Variable	HR [95% CI]	*p* value
LVEF (%)	1.04 [0.97–1.1]	0.253
Smoking habit before CRP (%)	0.35 [0.11–0.96]	0.048
Total cholesterol before CRP (mg/dL)	0.96 [0.9–1.02]	0.181
Mean change in total cholesterol (mg/dL)	0.94 [0.89–0.98]	0.004
HDL-C before CRP (mg/dL)	1.15 [1.07–1.23]	<0.001
LDL-C before CRP (mg/dL)	0.96 [0.9–1.03]	0.245
Mean change in LDL-C (mg/dL)	0.94 [0.89–0.99]	0.014
Lipoprotein (a) (mg/dL)	1.01 [1–1.02]	0.038
% change in IPAQ (per 100 METS/week)	1 [0.96–1.04]	0.924

Abbreviations: CRP, cardiac rehabilitation program; HDL-C, high-density lipoprotein cholesterol; IPAQ, International Physical Activity Questionnaire; LDL-C, low-density lipoprotein cholesterol; LVEF, left ventricular ejection fraction; METS, metabolic equivalents.

## 4. Discussion

When evaluating lipid parameters in patients with high cardiovascular risk, focus traditionally centers on markers including LDL-C, or more recently, lipoprotein (a). HDL-C often receives less attention, largely due to its complex relationship with cardiovascular risk and the limited evidence supporting drugs that effectively elevate HDL-C levels while concurrently reducing cardiovascular events. However, HDL-C remains a potential biomarker for cardiovascular risk, and achieving target HDL-C levels following ACS remains challenging for many patients [7]. Our study highlights the benefits of an exercise-based Phase 2 CRP following STEMI or OMI, which can not only significantly improve lipid profiles but also yields a modest but significant increase in HDL-C. By the end of Phase 2 CRP, a higher proportion of patients achieved normal HDL-C levels, although nearly half of the cohort exhibited low HDL-C levels despite the intervention. Factors such as previous smoking habits and substantial reductions to total cholesterol and LDL-C are associated with an HDL-C increase during Phase 2 CRP. Conversely, higher baseline levels of HDL-C and lipoprotein (a) predict failure to increase HDL-C.

### 4.1 HDL-C and Cardiovascular Risk

HDL-C consists of complex circulating particles with multiple subspecies that vary in lipid and protein composition. Specifically, HDL-C accounts for 25–30% of the circulating proteins responsible for lipid transport in the bloodstream. Although apolipoprotein AI (Apo AI) constitutes approximately 70% of its mass, the HDL-C proteome also includes a diverse array of other apolipoproteins and various enzymes [21, 22]. HDL-C is often referred to as “good cholesterol” due to its role in reverse cholesterol transport, a process through which excess cholesterol is removed from peripheral tissues and transported back to the liver for excretion or reuse. Additionally, HDL exerts various anti-inflammatory, antioxidant, antidiabetic, and antithrombotic functions [23, 24, 25, 26, 27]. Consequently, HDL-C has been traditionally described as a biomarker inversely associated with the risk of atherosclerotic cardiovascular disease events.

The investigation into the role of HDL-C in cardiovascular risk began in the 1960s [28], but gained significant momentum from the 1980s onward, following studies highlighting the adverse implications of low levels of this lipoprotein [29, 30]. Subsequent research, including the Framingham Study, corroborated these initial observations [31, 32, 33]. In both the Myocardial Ischemia Reduction with Aggressive Cholesterol Lowering (MIRACL) and Clinical Outcomes Utilizing Revascularization and Aggressive Drug Evaluation (COURAGE) trials, low HDL-C levels in patients with ACS and stable CAD were associated with increased risk of adverse cardiovascular events, and this effect was evident across the entire range of the observed LDL-C levels [10, 11]. Recent studies have also explored on the harmful effects of a high triglyceride/HDL-C ratio, indicative of residual cardiovascular risk even under conditions with optimal LDL-C levels [34, 35].

In recent years, various studies have suggested that extremely high levels of HDL-C can also adversely affect cardiovascular risk. Van der Steeg and colleagues showed that very large HDL-C particles were associated with an increased risk of CAD when apoA-I and apoB levels remained stable [36]. Building on this work, Madsen *et al*. [37] identified an inverse relationship between HDL-C and coronary risk even in patients with LDL levels below 60 mg/dL, while also noting a U-shaped curve between HDL-C and all-cause mortality. This paradoxical effect, where individuals with very low or very high levels of HDL-C experience higher mortality rates, is currently being investigated.

### 4.2 Pharmacological Options to Increase HDL-C

The premise that increasing HDL-C levels could offer cardiovascular protection has motivated numerous clinical trials. Despite these efforts, the outcomes of these trials have not demonstrated significant benefits associated with the interventions aimed at increasing HDL-C levels.

For example, the AIM-HIGH (Atherothrombosis Intervention in Metabolic Syndrome with Low HDL/High Triglycerides and Impact on Global Health Outcomes) trial included 3.414 patients treated with simvastatin +/– ezetimibe. This study compared the addition of niacin to a placebo. Although niacin treatment led to an improved lipid profile with an increase in HDL-C (+25%), a decrease in triglycerides (–28.6%), and a reduction in LDL-C (–12%), there was no clinical benefit from the niacin intervention [38]. Similarly, the HPS2-THRIVE (Heart Protection Study 2-Treatment of HDL to Reduce the Incidence of Vascular Events) trial included 25.673 adults who were randomized to receive 2 grams of extended-release niacin and 40 mg of laropiprant versus placebo. Despite improvements in lipid profiles analogous to those observed in the AIM-HIGH trial, this intervention did not significantly effect on the incidence of major cardiovascular events [39].

Fibrates are lipid-modifying agents known to reduce triglyceride levels and increase HDL-C. Although the VA-HIT (The Veterans Affairs HDL Intervention Trial) trial showed that gemfibrozil significantly reduced cardiovascular events in secondary prevention [40], other fibrates have not shown significant cardiovascular benefits despite improvements in lipid profiles [41, 42].

Cholesterol ester transfer protein (CETP) is predominantly secreted by the liver and adipose tissue and is primarily associated with high-density lipoproteins. CETP facilitates the transfer of cholesterol esters from HDL-C to triglyceride-rich lipoproteins such as VLDL and LDL. Although several CETP inhibitors have been evaluated in clinical trials, they have not shown a significant reduction in cardiovascular risk, despite increasing HDL-C levels [43, 44, 45]. For instance, anacetrapib resulted in a significant increase to HDL-C (+104%) and a decrease in LDL-C (–41%), along with a reduction in cardiovascular events, but its development was discontinued due to safety concerns related to its accumulation in adipose tissue [46]. Currently, obicetrapib has completed phase II with a substantial reduction in LDL-C and increase in HDL-C, but it is still in phase III development [47].

These discouraging results from trials targeting HDL-C can be attributed to several factors. First, the heterogeneity of HDL-C subfractions may have varying impacts on cardiovascular risk, which complicates the interpretation of overall HDL-C levels [13, 15, 48]. Additionally, the focus on measuring the absolute levels of HDL-C may overlook its functional capacities, which include cholesterol efflux capacity, antioxidant properties, anti-inflammatory effects, and immune system regulatory activities [14, 49]. Another significant issue is the negative influence of oxidative modifications by free radicals on HDL-C particles. When HDL-C is oxidatively modified, it can assume pro-inflammatory properties, which could negate its cardiovascular benefits [50]. Further research and clinical trials should clarify whether pharmacological increases in HDL-C can improve clinical outcomes.

### 4.3 Cardiac Rehabilitation and HDL-C

Major scientific societies recommend the inclusion of ACS patients in CRP to improve prognosis, quality of life, and control of cardiovascular risk factors [2, 3, 4]. According to our findings, an exercise-based Phase 2 CRP following STEMI/OMI has a beneficial effect on the lipid profile, including a slight but significant increase in HDL-C, in line with previous studies [51, 52, 53]. Moreover, it has been demonstrated that a CRP not only increases HDL-C levels but also improves HDL function, specifically its cholesterol efflux capacity [54, 55].

CRP can be performed either as a center-based cardiac rehabilitation (CBCR) or cardiac telerehabilitation (CTR). Both forms of intervention have demonstrated positive results with notable improvements to lipid profiles [53, 56]. Dalli Peydró *et al*. [57] recently compared an 8-week CBCR program with a 10-month CTR program. Their 10-month CTR program increased physical activity and peak VO2, improved the lipid profile and quality of life, and along with increased adherence when compared to a CBCR program [57], similar to a previous study [58]. The effect of CTR on improving maximal oxygen consumption appears to be dependent upon the duration and similar to CBCR [59].

The improvement of lipid profile during CRP, specifically the increase in HDL-C levels, can be attributed to multiple factors. While some drugs are specifically designed to increase HDL-C, commonly used lipid-lowering medications that are primarily aimed at reducing LDL-C can also contribute to increased HDL-C levels. Statins, ezetimibe, and PCSK9 inhibitors are notable examples of such drugs, each capable of modestly enhancing HDL-C levels alongside their primary effects [60, 61, 62]. However, HDL-C increase during CRP can be explained by other factors.

Several studies have shown that physical exercise reduces body fat while improving blood pressure and lipid profiles, thereby lowering cardiovascular risk [63, 64]. Specifically, aerobic exercise appears to have a notable impact on reducing total cholesterol and LDL-C and increasing HDL-C levels, whereas short, high-intensity exercise seems to have a lesser impact on lipid profile [65, 66, 67]. These effects may be sex dependent, since women may benefit from low to moderate intensity aerobic activity, while men might require more intense physical activity to achieve the same benefits [68, 69].

Diet also exerts a significant influence on both HDL-C and lipid profiles. Particularly the combination of high-intensity training with dietary intervention has been shown to significantly increase HDL-C levels [67]. HDL-C can also be increased by the intake of omega-3 fatty acids, adherence to a Mediterranean diet, and weight loss associated with dieting [70, 71, 72]. Finally, mental health and well-being have also been suggested to influence lipid profiles, including higher levels of HDL-C [73, 74].

In our CRP, a comprehensive approach incorporating multiple strategies played a pivotal role in enabling patients to raise their HDL-C levels. Lipid-lowering therapy optimization was performed to achieve target LDL-C levels [75], which has the secondary benefit of modestly increasing HDL-C. Additionally, both ambulatory and hospital-based physical training programs were tailored to individual patient needs, resulting in high levels of physical activity by the end of Phase 2. Dietary modifications were also integral, with a specific emphasis on the Mediterranean diet which participants adhered to well. The success in smoking cessation among previous smokers further contributed to the overall positive lipid profile outcomes. Moreover, patients reported an improvement in quality of life and psychological well-being. These multifaceted interventions collectively underscore the effectiveness of the CRP in improving not only the lipid profiles but also the broader health parameters of the participants.

Even though our study demonstrated an increase in HDL-C levels and in the proportion of patients reaching normal HDL-C values, some individuals failed to increase HDL-C and nearly half of the cohort continued to exhibit low HDL-C levels despite the intervention. Identifying these patients is crucial as it could allow for the implementation of more intensive non-pharmacological interventions or targeted pharmacotherapy. The effectiveness of such targeted approaches could be further validated if ongoing clinical trials yield favorable evidence. This stratified approach ensures that all patients receive the most effective and personalized treatment strategies to optimize their cardiovascular health outcomes.

Higher baseline levels of HDL-C were predictive of a lesser likelihood of increasing HDL-C following CRP, likely because patients with initially high levels have less potential for further improvement. This phenomenon, observed in the VOYAGER (an indiVidual patient meta-analysis Of statin therapY in At risk Groups: Effects of Rosuvastatin, atorvastatin, and simvastatin) meta-analysis, highlights that baseline HDL-C levels can predict the extent of increase induced by statin therapy [76]. Treatment with rosuvastatin 10 mg o.d. induced a 11.4% increase in HDL-C among patients in the lowest HDL-C quintile (<39 mg/dL) but only a negligible change (–0.2%) in those in the highest quintile (>59 mg/dL) [76]. Similar results have also been described with the use of fibrates [77], suggesting a ceiling effect where potential for further HDL-C increases are diminished as baseline levels increase.

Higher baseline levels of lipoprotein (a) also seem to hinder HDL-C increases during CRP like interventions. This relationship is underscored by findings that lipoprotein (a) positively influences the HDL-C production pathway in hepatocytes, suggesting a regulatory role in HDL-C metabolism [78]. Additionally, some studies have indicated that lipoprotein (a) levels are related to HDL-C levels in certain populations, indicating a potentially broader interaction between these lipoproteins [79, 80]. However, further research is necessary to fully understand the mechanisms by which lipoprotein (a) might inhibit HDL-C increases in the context of CRP interventions.

Significant reductions in total cholesterol and LDL-C after CRP participation were associated with more pronounced increases in HDL-C. This correlation likely indicates greater overall response to lipid-lowering treatment [81], especially considering that some lipid-lowering drugs aimed at reducing LDL-C also exert a modest positive effect on HDL-C [60, 61, 62]. Furthermore, a history of smoking was linked to an increase in HDL-C levels after CRP. This finding is largely attributable to the high cessation rate during Phase 2 of CRP, where approximately 90% of participants quit smoking. Smoking is consistently associated with lower HDL-C levels [82], and smoking cessation has been shown to significantly increase HDL-C levels [83].

Taken altogether, our results indicate that CRP can improve HDL-C levels, along with other aspects of the lipid profile and several patient-centered outcomes. However, it is noteworthy that nearly half of the participants did not achieve normal HDL-C levels. Our analysis also identifies reliable predictors for the inability to increase HDL-C, which include baseline lipid profile variables such as HDL-C and lipoprotein (a), dynamics of total cholesterol and LDL-C, as well as clinical variables including smoking habits. These predictive factors can be utilized to identify patients who are less likely to reach target HDL-C levels. Recognizing these individuals allows for a more tailored approach, where more intensive interventions or emerging therapies can be applied as they become available. This individualized strategy enhances the potential for achieving optimal lipid management and improving overall cardiovascular health outcomes within the CRP framework.

### 4.4 Limitations

Our study has several limitations that should be considered. First, it derives from a single-center cohort with a relatively low number of patients, so our results may not be generalizable to other populations. Second, we only included patients presenting with STEMI or OMI, so our cohort is not representative of the whole spectrum of ACS (e.g., non-ST-elevation ACS or unstable angina). Third, due to the observational nature of our study, no control group was available, and several biases, such as selection and information bias, couldn’t be excluded. For all these reasons, the findings of our research should be considered exploratory. Future studies should increase sample size and improve the population representation by including all ACS subtypes, incorporate other centers for external validation, and ideally incorporate a control group to confirm the effectiveness of the CRP intervention.

## 5. Conclusions

Our exercise-based Phase 2 CRP achieved modest but significant increases to patient HDL-C levels. However, a significant percentage of participants did not reach the target HDL-C levels described in clinical practice guidelines. Our observations indicate that patients who cease smoking and achieve significant reductions in total cholesterol and LDL-C following CRP are more likely to experience increases in HDL-C. These results suggest that intensive lipid-lowering therapy, structured exercise regimens, and smoking cessation during CRP can have significant implications for increasing HDL-C levels. Conversely, patients with higher baseline levels of HDL-C and lipoprotein (a) prior to CRP are less likely to achieve increased HDL-C levels. Further studies are needed to confirm these findings and to provide specific guidance on HDL-C management. Given the complex relationship between HDL-C and cardiovascular risk, it is essential to determine the clinical significance of HDL-C changes during CRP and their correlation with long-term outcomes. This will help clarify whether modifications in HDL-C brought about by CRP can substantially impact patient prognosis and guide future therapeutic strategies.

## Availability of Data and Materials

The data used and/or analyzed during the current research are available from the corresponding author on reasonable request. The data are not publicly available due to ethical restrictions.
